# Progressing the Development of a Collaborative Metareasoning Framework: Prospects and Challenges

**DOI:** 10.3390/jintelligence12030028

**Published:** 2024-03-01

**Authors:** Beth H. Richardson, Linden J. Ball

**Affiliations:** School of Psychology & Humanities, University of Central Lancashire, Preston PR1 2HE, UK; lball@uclan.ac.uk

**Keywords:** metareasoning, metacognitive monitoring, metacognitive control, collaboration, uncertainty, reasoning, teams, alignment, intersubjectivity, language

## Abstract

Metareasoning refers to processes that monitor and control ongoing thinking and reasoning. The “metareasoning framework” that was established in the literature in 2017 has been useful in explaining how monitoring processes during reasoning are sensitive to an individual’s fluctuating feelings of certainty and uncertainty. The framework was developed to capture metareasoning at an *individual* level. It does not capture metareasoning during collaborative activities. We argue this is significant, given the many domains in which team-based reasoning is critical, including design, innovation, process control, defence and security. Currently, there is no conceptual framework that addresses the nature of collaborative metareasoning in these kinds of domains. We advance a framework of collaborative metareasoning that develops an understanding of how teams respond to the demands and opportunities of the task at hand, as well as to the demands and opportunities afforded by interlocuters who have different perspectives, knowledge, skills and experiences. We point to the importance of a tripartite distinction between “self-monitoring”, “other monitoring” and “joint monitoring”. We also highlight a parallel distinction between “self-focused control”, “other-focused control” and “joint control”. In elaborating upon these distinctions, we discuss the prospects for developing a comprehensive collaborative metareasoning framework with a unique focus on language as a measure of both uncertainty and misalignment.

## 1. Introduction

The concept of “metareasoning” refers to the metacognitive processes that *monitor* and *control* the core cognitive operations associated with reasoning, which are often referred to as “object-level” processes (cf. Nelson and Narens 1990). These processes include those focused on interpreting and understanding available information as well as drawing inferences from it and making decisions about courses of actions to take (see Ackerman and Thompson 2017, 2018). Object-level processes can be distinguished from monitoring and control processes that function at a metalevel. At this metalevel, continual monitoring is essential to evaluate the effectiveness of object-level processes in progressing toward good outcomes, including sound inferences, and convincing decisions. Metacognitive monitoring is usually experienced subjectively in terms of one’s awareness of a shifting state of certainty regarding how effectively a process is unfolding, as well as one’s perception of how good a resulting outcome seems to be (Ackerman and Thompson 2017, 2018).

Like monitoring processes, control processes similarly function at a metalevel, but serve different fundamental purposes, including: (i) the allocation of essential cognitive resources, such as attention and working memory, to object-level processes; (ii) the termination of object-level processing that is failing to deliver a suitable outcome; (iii) the initiation of new object-level processing when it seems necessary to do so. It is also important to note that, during any reasoning task, there will be a continual interaction between monitoring and control processes to ensure that object-level cognition is maintained and progresses until an outcome is reached. If people feel confident about the efficacy of their object-level processing, then they will be likely to continue pursuing a particular course of action, whereas, if they reach a point where they have low confidence in what they are doing, then they might change their current strategy, ask for help or decide to give up on the task entirely (e.g., see Law et al. 2022 for an in-depth discussion of people’s giving-up behaviour on reasoning tasks).

The “metareasoning framework” that has been developed by Ackerman and Thompson (2017, 2018) represents a key milestone in recent efforts to explain many findings relating to the processes that monitor and control object-level reasoning. This metareasoning framework captures the way in which monitoring processes are sensitive to fluctuating feelings of certainty and uncertainty that are experienced by a reasoner during task performance, as well as the way in which different control processes are evoked in response to heightened levels of certainty or uncertainty. It is important to emphasise, however, that this framework was developed to explain metareasoning as it arises in individual reasoners rather than in groups of reasoners who are collaborating to undertake a joint reasoning task, as might arise, for example, in team problem solving or decision making. The focus of the metareasoning framework on *individuals* clearly raises the interesting question of whether the framework—or at least some key aspects of it—can be extended to capture the nature of *collaborative* metareasoning. Addressing this question provides a key motivating rationale for the present paper, which aims to explore the prospects and challenges for progressing the development of a collaborative metareasoning framework.

We begin by highlighting the importance of understanding collaborative metareasoning for theoretical advancement, especially in relation to the monitoring and control processes that arise during team reasoning, problem solving and decision making in real-world contexts. We next overview Ackerman and Thompson’s (2017, 2018) influential metareasoning framework, which is focused on individual reasoners, and we consider potential ways in which this framework can inform an understanding of collaborative metareasoning. In overviewing Ackerman and Thompson’s (2017, 2018) conceptual ideas, we emphasise how their framework focuses primarily on self-report measures of fluctuating levels of certainty and uncertainty. When it comes to an analysis of *collaborative* metareasoning, however, we argue that there is an opportunity to gain more direct access to monitoring and control processes through a detailed analysis of the individual and joint language that arises in team reasoning contexts. As such, our paper progresses toward a detailed consideration of current research on the metacognitive monitoring and control processes that are critical to the occurrence of effective *dialogue* during joint action (Pickering and Garrod 2021; see also Gandolfi et al. 2023). We argue that an analysis of team dialogue has the potential to pave the way toward a rich understanding of collaborative metareasoning that has so far been starkly absent in the literature.

Throughout our paper, we continually highlight an important tripartite distinction that was articulated in the context of metareasoning by Richardson et al. (2024; see also Pickering and Garrod 2021) between “self-monitoring” (i.e., an individual’s perception of their own performance), “other monitoring” (i.e., an individual’s perception of the performance of others) and “joint monitoring” (i.e., the unified perception of collective performance). Furthermore, in this paper, we also introduce a parallel tripartite distinction that relates to control processes, which is captured by the notions of “self-focused control” (i.e., an individual’s decisions about how to progress or terminate their own reasoning), “other-focused control” (i.e., an individual’s decisions about how to control the performance of others) and “joint control” (i.e., the unified control of decisions regarding how to advance or terminate collective performance). Our detailed discussion of these distinctions culminates in a consideration of the opportunities for developing a comprehensive collaborative metareasoning framework and the associated challenges.

## 2. The Importance of Understanding Collaborative Metareasoning

Most existing research on metacognitive monitoring and control has been undertaken with the aim of enhancing educational outcomes (e.g., Cromley and Kunze 2020; Hacker et al. 2009; Perry et al. 2019) and has, therefore, focused on object-level cognition that is related to comprehending and remembering in learning contexts. There has, however, been a limited but nevertheless long-standing interest in metacognitive monitoring and control processes in other areas of research, particularly in situations involving reasoning (e.g., Ackerman and Beller 2017; Quayle and Ball 2000; Thompson et al. 2011, 2013) and problem solving (e.g., Ackerman 2014; Ackerman and Zalmanov 2012; Metcalfe and Wiebe 1987; Topolinski and Reber 2010). Although such research has tended to be almost exclusively laboratory-based, more recent studies have broadened their focus to investigate metareasoning outside of the laboratory. One example is a study by Pervin et al. (2015) that analysed an extensive set of tweets to reveal how the use of hashtags in Twitter appears to be driven by an individual’s metacognitive experiences relating to cognitive load and confusion. Another example is a study by Roberts (2017), which used a community sample to examine the role of metacognitive monitoring and control in the acquisition of skills relating to effective web search and evaluative reasoning. Yet another example is a study by Zion et al. (2015) examining the effects of metacognitive support on metareasoning within an online discussion forum in the context of computer-supported inquiry.

Despite such exceptions, however, there remains a paucity of studies of metareasoning in relation to individuals working in real-world situations, and this dearth of research is likewise seen in relation to metareasoning in collaborating teams engaged in professional work-based practices. The relative absence of studies examining the nature of metareasoning processes during collaboration is unfortunate given that effective team-based performance is so critical in many domains. A few examples include contexts relating to design, innovation, medical diagnosis, security, defence and emergency response.

These latter domains all depend upon collaborative problem solving, reasoning and decision making operating effectively to achieve shared goals. For example, in high-risk defence and security situations, a failure to coordinate team decision making can have highly negative downstream consequences, including loss of life and damage to infrastructure. Recently, the media reported on inherent inadequacies in the response by emergency teams during the 2017 Manchester Arena attack. Three key failures were identified: (i) communication was ineffective between teams; (ii) coordination both within and between teams was weak, as reflected in a failure to identify priorities and resources; (iii) shared situational awareness was poor in that plans of action were not clearly understood. These failures were all ones relating to inadequate metareasoning. It has been argued (see Saunders 2022) that, with better team collaboration, the negative outcome of the Manchester Arena attack could have been reduced.

In emergency response situations such as those arising in the Manchester Arena attack, metacognitive monitoring processes need to be attuned to the subtleties and complexities of uncertainty at both a *personal* and *interpersonal* level. In the latter case, uncertainty will typically manifest in the language that arises in team dialogue and cross-team communication, such as the use of tentative language and hedge words (e.g., “maybe”, perhaps” and “possibly”). Metacognitive control processes, on the other hand, need to affect dynamic strategy change in a coordinated manner (i.e., mutually agreed between members, either tacitly or explicitly) to ensure that successful decisions are made. Within this complex, dynamic communication context there will also be a variety of situational factors that are likely to have an impact on object-level and metalevel processing at both an *inter*personal and *intra*personal level, such as whether team members get on well with one another and whether collaborators are perceived to be competent. Recent studies of team-based emergency responding have corroborated the critical role of metacognitive monitoring and control in ensuring effective situation awareness between team members and enhanced team decision making (see Hamilton et al. 2017).

The example of decision making in the context of teams engaged in emergency responding serves to illustrate the potentially critical role played by metareasoning during real-world collaboration. The same kinds of monitoring and control processes that arise in team-based emergency responding will presumably also be involved in any jointly executed activity that involves the attainment of some common goal, whether this involves making a shared decision regarding a course of action to take or generating an agreed approach concerning how to solve a complex problem. Nevertheless, a clear conceptualisation of the metacognitive monitoring and control processes that underpin collaborative problem solving, reasoning and decision making is absent from the literature and, we contend, is in pressing need of development. To advance the formulation of such a collaborative metareasoning framework, we next review the existing literature on metareasoning at the level of the individual, before then taking this useful body of knowledge forward and extending it to contexts involving collaboration.

## 3. The Metareasoning Framework

**Figure 1:**
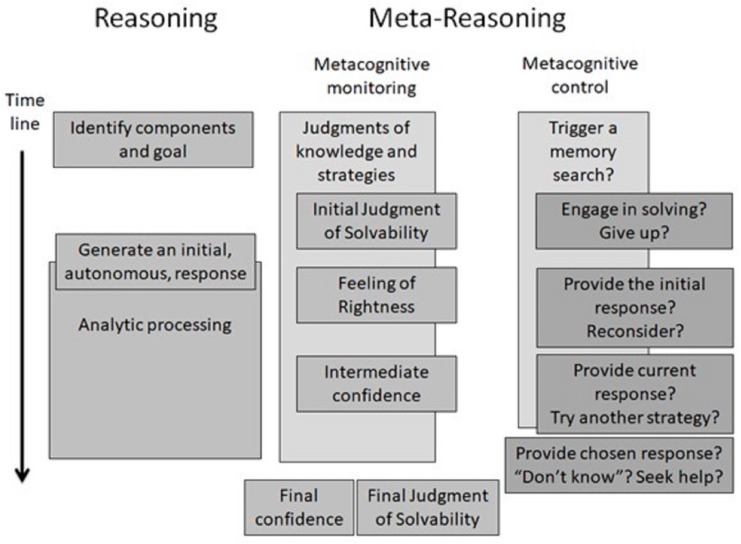
The approximate time course of reasoning and metareasoning processes as captured in Ackerman and Thompson’s (2018) metareasoning framework. Copyright (©2018) from “Meta-reasoning: Shedding meta-cognitive light on reasoning research” by Ackerman and Thompson (2018). [Permission to reproduce has been sought from Taylor and Francis Group, LLC, a division of Informa plc].

The metareasoning framework, proposed by Ackerman and Thompson (2017, 2018; see Figure 1), has sparked considerable research interest in relation to metacognitive monitoring and control processes in domains that involve problem solving, reasoning and decision making. Figure 1 displays the approximate time course of object-level reasoning as well as corresponding metareasoning processes (Ackerman and Thompson 2017, 2018). The left side of the figure represents the object-level processes involved in reasoning, whilst the middle column depicts the associated monitoring processes. Monitoring processes reflect a reasoner’s subjective evaluation of the probability of success or failure in relation to a given task or problem (e.g., their confidence in the unfolding process), which can occur before, during or after object-level processing.

Crucially, monitoring processes continually track fluctuating levels of certainty and uncertainty related to ongoing task performance or solution success. Within this metareasoning framework, monitoring processes are viewed as happening in the background and as having the capacity to “trigger” control processes. The right-hand column of Figure 1 depicts such control processes, which serve to allocate and redistribute resources in response to the outcome of ongoing monitoring. For example, if intermediate confidence is high, individuals will be likely to continue with their current course of action. In contrast, if they experience low–intermediate confidence or a feeling of uncertainty, they may activate control processes to switch their current strategy or, alternatively, they may give up.

The object-level processes that are shown in Figure 1 include ones that are associated with problem understanding and goal identification, as well as ones that generate an initial, autonomous response to the task at hand or that involve reasoning about the task analytically. Moreover, the object-level reasoning that is portrayed in Figure 1, whereby intuitive processing is followed by analytic processing, aligns with a “dual-process” architecture that is based on “default-interventionist” principles (Evans and Stanovich 2013a, 2013b). According to such an architecture, reasoning is considered to involve two qualitatively distinct types of processes, referred to as Type 1 and Type 2. Type 1 processes are viewed as being intuitive, heuristic and associative in nature, and are defined in terms of being relatively undemanding of working-memory resources as well as autonomous (running to completion whenever they are cued). Correlated features of Type 1 processes are that they tend to be high-capacity, rapid, nonconscious and capable of running in parallel. In contrast, Type 2 processes are reflective, deliberate, analytic and controlled, and are defined in terms of requiring working memory resources and having a focus on hypothetical thinking. Correlated features of Type 2 processes include their tendency to be slow, capacity-limited, serial and conscious. Furthermore, Type 2 processes are less prone to biases in comparison to Type 1 processes, although they are not invulnerable to them (e.g., see Evans 2018; Evans and Stanovich 2013a, 2013b), which may arise, for example, from the application of inadequate or inappropriate analytic operations, referred to as “defective mindware” (e.g., Stanovich 2018).

Assuming a default-interventionist version of the metareasoning framework, one key function of metacognitive monitoring is to trigger a strategic shift from default Type 1 to analytic Type 2 processing so that analytic processes can intervene to determine the accuracy of the default response. To illustrate this shift from Type 1 to Type 2 processing, consider the bat-and-ball problem that is one of the items in the Cognitive Reflection Test (Frederick 2005; Kahneman and Frederick 2002). This problem reads as follows: “A bat and a ball cost $1.10 in total. The bat costs $1.00 more than the ball. How much does the ball cost?” This problem might seem easy at first sight, yet many highly intelligent individuals generate an incorrect solution (e.g., Campitelli and Gerrans 2014; Pennycook et al. 2016; Stupple et al. 2017). Incorrect answers are attributed to a dominant, intuitive Type 1 response, whereby the answer that comes to mind rapidly and easily (i.e., 10 cents) is, in fact, wrong. When producing intuitive but incorrect responses, individuals may experience a low feeling of rightness (Thompson et al. 2011, 2013; see also Bago and De Neys 2017), which for some people will trigger a shift to Type 2 analytic reasoning, such that they might then be able to determine the correct answer.

Although Ackerman and Thompson’s (2017, 2018) metareasoning framework is couched in dual-process terms that capture default-interventionist principles, they stress that the logic of monitoring processes, such as those that register a “feeling of rightness” (see Figure 1), also extends to single-process reasoning theories that do not propose two types of processing (e.g., Kruglanski and Gigerenzer’s 2011, “Unified Theory of Judgment”). Moreover, the logic also pertains to theories of reasoning that propose the existence of multiple, parallel processes rather than sequential ones (e.g., see Bago and De Neys 2017). Whatever model of object-level reasoning one favours, it remains important to understand when, why and how people engage in more deliberate, reflective thinking. This, in turn, speaks to the importance of gaining insight into the way that people monitor rapid, initial answers, regardless of the type of reasoning mechanisms that are proposed to underlie their generation. The flexibility of the metareasoning framework to accommodate different theoretical perspectives on object-level reasoning is certainly a key strength.

## 4. Research Questions in Metareasoning Research

Ackerman and Thompson’s (2017, 2018) metareasoning framework provides a rich and productive theoretical foundation for research on reasoning and metareasoning. Nevertheless, the framework is primarily just a starting point for further studies and conceptual developments in this area. The need for an extensive amount of further research was directly acknowledged by Ackerman and Thompson (2017), who presented a series of research questions that are now increasingly being addressed by several international teams. Among these research questions are ones that are concerned with whether reasoners have a degree of insight into the sources of certainty that affect their judgments, whether metareasoning processes are shaped by culture and whether reasoning performance can be enhanced through interventions that are targeted at improving metareasoning skills.

As we have also noted above, a critical area of development for the metareasoning framework relates to its extension to capture collaborative metareasoning rather than just individual metareasoning. Little research has been conducted on this topic to date, leaving many questions wide open for further research and conceptual advancement. However, before we move on to explore some of the relevant challenges and opportunities relating to the development of an understanding of collaborative metareasoning, we first describe some important ways in which Ackerman and Thompson’s current framework has recently been extended. Doing this will allow us to introduce important ideas that will help inform our subsequent discussion of the complex issues that a collaborative metareasoning framework will need to tackle if it is to provide even the semblance of an account of how metareasoning operates in team contexts.

### 4.1. The Underpinning Basis of Metacognitive Certainty and Uncertainty

One critically important research question that needs to be addressed for an advancement of metareasoning—whether in situations that involve individual or team reasoning—concerns the underpinning basis of the fluctuating feelings of certainty and uncertainty that are experienced during task-based processing. One of the most pervasive cues that has been suggested to elicit such feelings is the perceived *ease* of task-oriented processing, which is a cue that is typically referred to as “processing fluency” (e.g., Alter and Oppenheimer 2009; Unkelbach and Greifeneder 2013). In essence, an answer or solution that comes to mind quickly and easily (i.e., with high processing fluency) gives rise to a strong feeling of rightness as well as to a heightened assessment of final confidence (Ackerman and Zalmanov 2012; Thompson and Morsanyi 2012; Thompson et al. 2013).

Processing fluency is usually indexed as the time that has elapsed from when a problem is displayed until a participant provides their response, with reasoning and problem-solving tasks typically showing a negative correlation between response time and metacognitive confidence judgments, reflecting the fact that easy items are processed more quickly than difficult items (e.g., Ackerman 2014; Ackerman and Zalmanov 2012; Baars et al. 2013). The influence of processing fluency on confidence has also been observed in studies of metacognition and memory. For example, Undorf and Erdfelder (2015) found that processing fluency—measured in terms of self-paced study time for to-be-remembered items—partially mediated the effect of item ease/difficulty on the judged probability of recalling the target (indexing “judgment of learning”). It is also important to note that the frequently observed association between processing fluency and confidence seems not merely to be correlational, but rather reflects a causal mechanism, whereby the ease of information processing directly induces a high degree of confidence in the correctness of a response (e.g., Topolinski and Reber 2010).

Other recent research on people’s metacognitive judgments in relation to memory tasks has been influential in shedding further light on the sources of information that people use in addition to processing fluency when making metacognitive judgments. This research has been especially valuable in revealing how people appear to integrate *multiple* cues that stem from the same stimulus item when making judgments of learning (e.g., Undorf and Bröder 2021; Undorf et al. 2018). In such studies, Brunswik’s lens model (e.g., see Kaufmann 2022) has been used to assess the overall validity of confidence judgments based on a combined set of cues. As Ackerman (2023) notes, however, this research leaves open important questions regarding the relative weights of such cues and how these weights change across task designs (e.g., different instructions) and population characteristics (e.g., different levels of prior knowledge). Ackerman (2023) presents a novel statistical approach, the “Bird’s-Eye View of Cue Integration” (BEVoCI) methodology, which she argues can reveal not only the cues that determine metacognitive judgments and task success, but also the relative weights of these cues and their malleability across task designs and populations.

Ackerman’s (2023) experiments using the BEVoCI method provide a wealth of findings regarding the multiplicity of weighted cues that underpin confidence judgments and solution accuracy in problem solving as well as the way in which these cues can dissociate in determining these outcome measures. Ackerman (2023) reports that people’s judgments of confidence are consistently oversensitive to processing fluency (as indexed by response time), which is a phenomenon that is well-established in the metareasoning literature (e.g., Ackerman and Zalmanov 2012). We see considerable value in cue-integration methods for advancing an understanding of the information that people draw upon when providing confidence judgments regarding final solutions to reasoning problems. Nevertheless, whether such methods can also be successfully extended to predict the cues that underpin judgments of *intermediate* confidence, such as people’s feeling of rightness, is yet to be determined.

### 4.2. Methods for Eliciting Judgments of Metacognitive Certainty and Uncertainty

A further, important research question concerning metareasoning in both individual and team contexts relates less to the issue of the cues that drive confidence judgments, and more to the question of how best to elicit confidence judgments from participants in the first place. As can be seen in Figure 1, confidence judgment can be elicited from participants at various time points, including, for example, initial judgments of solvability, intermediate feelings of rightness or confidence and final judgments of confidence or solvability. Our primary focus in this paper is on the best way to elicit *intermediate* confidence judgments at various points during the reasoning process, as this represents a critical measurement issue for understanding metacognitive monitoring in both individual and team reasoning contexts.

In problem-solving research, the traditional method that is used to obtain ephemeral, subjective judgments of certainty from a reasoner necessitates intermittently probing them for a rating of their current degree of confidence regarding the likelihood of finding a solution to a given problem. In research with *individual* problem solvers, this kind of metacognitive probe has often been described as indexing a reasoner’s current “feeling of warmth”; that is, their sense that a solution is imminent (Metcalfe 1986; Metcalfe and Wiebe 1987; see also Hedne et al. 2016; Kizilirmak et al. 2018; Laukkonen and Tangen 2018). However, translating a fleeting feeling of warmth into a response on a numerical or categorical scale is a challenging requirement for participants who are midtask, and who therefore have simultaneously to maintain primary task performance while also making known their current metacognitions about likely task success.

It is also important to consider the possibility that the temporary cessation of primary task processing while generating a metacognitive judgment might have a *reactive* effect on ongoing object-level reasoning, changing its natural dynamics, trajectory, and outcome. There is only a relatively limited body of experimental evidence that has directly addressed the reactive effect of probing confidence on individual object-level reasoning, with much of the research tending to examine the reactivity of eliciting *retrospective* confidence judgments on decision time and performance accuracy in studies involving repeated trials. Intriguingly, the relevant studies that have been conducted to date provide evidence both for the idea that metacognitive confidence judgments are reactive (e.g., Baranski and Petrusic 2001; Bonder and Gopher 2019; Double and Birney 2017, 2018, 2019a; Lei et al. 2020; Petrusic and Baranski 2003; Schoenherr et al. 2010), as well as against this idea (e.g., Ackerman 2014; Ackerman and Goldsmith 2008; Ackerman et al. 2020).

We do not wish to be drawn into a detailed consideration of the potential reasons for the discrepant findings in the extant literature regarding the reactivity of confidence judgments, as the matter seems to be a long way from being resolved. Much more empirical work and conceptual development will be required to understand the conditions under which reactivity arises and the severity of its impact on performance, whether assessed in terms of processing time or outcome quality (for relevant discussion see Double et al. 2018; Double and Birney 2019b). In the absence of a solid empirical and theoretical foundation from which to make definitive predictions as to when and why some metareasoning studies show reactivity whilst others do not, we would argue that it seems judicious to conduct a parallel line of research that measures confidence using *alternative* methods. We also contend that this argument pertains as much, if not more, to team-based, rather than individual, reasoning situations. This is because reactivity arising from continually interrupting team members to elicit confidence judgments during their ongoing collaboration could impact not only the object-level processing of individual team members but also the whole team dynamic.

What, though, might alternative methods for eliciting confidence judgments entail such that they can provide a *nonreactive* measure of fluctuating certainty and uncertainty as an individual or team progresses from initial problem understanding toward final solution generation? In individual reasoning contexts, the use of think-aloud methods (e.g., Ericsson and Simon 1980), where solitary participants verbalise whatever is currently passing through their minds, may seem like a way forward to obtaining a nonreactive, dynamic index of fluctuating levels of confidence. This is because spoken language can provide an excellent source of rich information about a person’s changing states of uncertainty, which can be manifest through their use of hedge words (e.g., “maybe”, “perhaps” and “possibly”). Of course, the think-aloud method cannot be deployed to assess fluctuating uncertainty during team-based reasoning, as it is a technique that is only appropriate for use with individual reasoners, which immediately limits its utility. However, it may even be the case that the method is ill-suited to providing a valid, dynamic index of uncertainty in individual reasoning, given long-standing concerns about the potential for thinking aloud itself to have a reactive effect on object-level reasoning, potentially changing natural processing in profound ways (e.g., see Godfroid and Spino 2015).

The reactive effect of thinking aloud is exemplified by the phenomenon of “verbal overshadowing”. This arises when participants are asked to verbalise their thoughts while attempting problems whose solution discovery benefits from restructuring processes that give rise to feelings of “insight”, with these restructuring processes operating primarily at an unconscious level. Pioneering research by Schooler et al. (1993; see also Schooler and Melcher 1995) revealed that thinking aloud *hindered* the attainment of insightful solutions via unconscious restructuring processes. This effect appears to arise because the request to think aloud diverts the problem solver’s attention toward strongly activated and obvious aspects of the problem that can easily be verbalised but are irrelevant to its solution (e.g., Bowden et al. 2005). In addition, more weakly activated information that *is* critical for solution success, but which resides at a level below awareness, is unable to enter consciousness because it is blocked or overshadowed by the stronger and reportable—albeit misdirected—information (cf. Ball et al. 2015; Kershaw and Ohlsson 2004; Siegler 2000). Although this verbal overshadowing effect is not always found in studies of insight problem solving, Ball et al. (2015) have suggested that occasional failures to replicate may be attributable to methodological differences between studies.

Considering the potential reactivity that can be engendered through the deployment of the think-aloud method in metareasoning research, the question arises as to whether alternative and less reactive techniques are available to detect fluctuating states of uncertainty during ongoing reasoning. Some possible options that come to mind include the use of eye-gaze tracking (e.g., Ball 2013), pupillometry (e.g., Mathôt and Vilotijević 2022) and eye-blink rate (Paprocki and Lenskiy 2017), as well as physiological measures, such as skin conductance (Figner et al. 2019) and heart-rate variability (Forte et al. 2019). The challenge with these methods, however, is that they have not traditionally been used to pinpoint states of uncertainty during task performance, instead being more closely associated with measures of fluctuating cognitive workload, arousal, stress and fatigue. It may well be that these methods can be deployed in a way that can index states of uncertainty during reasoning, but we are not aware of research that has attempted to do this to date, and it might be that the challenges to do so are simply insurmountable.

Recently, however, one interesting method that has been successfully deployed to measure continuous changes in the feeling of warmth states during problem solving involves the use of a so-called “dynamometer” (Laukkonen et al. 2021). This involves acquiring a continuous measure of fluctuating hand-grip strength from participants who are instructed to use grip intensity to indicate changing feelings of perceived progress toward a solution to a problem that can typically be solved through an unconscious restructuring process. In Laukkonen et al.’s (2021) study, participants were instructed to squeeze the dynamometer more strongly to convey greater perceived progress, and they were also asked to give the dynamometer a full-strength squeeze if they had found the solution via insight and experienced an Aha! moment, or to release their grip quickly if they had reached the solution without an Aha! moment.

The dynamometer allowed Laukkonen et al. (2021) to acquire multiple data points per second in real-time in relation to the onset of insight experiences, additionally enabling them to map such metareasoning data to other measures (i.e., solution accuracy and solution confidence) to investigate convergent validity, which was found to be high. For example, “spikes” in the dynamometer converged with participants verbally reporting after the problem-solving trial that they had experienced an Aha! moment. We note, however, that Laukkonen et al. (2021) did not directly test for an absence of reactivity on problem-solving performance arising from the deployment of the dynamometer, which is a key limitation of their study. Nevertheless, they confirm that problem-solving success rates in their study were comparable or better than those observed for similar tasks in other studies of insight problem solving (i.e., Salvi et al. 2016; Webb et al. 2016, 2018), supporting an apparent lack of reactivity arising from participants having to use the dynamometer to register feelings of progress.

Overall, Laukkonen et al. (2021) suggest that the dynamometer may be a useful tool in any context where researchers are interested in continually measuring metacognition, such as in situations that involve problem solving and insight. The nonverbal nature of the dynamometer presumably makes it far less likely that it will interfere with the demands of primary task processing, such that it should have minimal reactive impact on natural object-level reasoning. In addition, the technique could presumably be utilised with multiple, collaborating problem solvers (each using a dynamometer), although the use of a hand-held device certainly limits the feasibility of it being deployed anywhere other than in relatively simple individual or collaborative reasoning and problem-solving situations. More complex problems of the type that arise in real-world contexts would require free hand movement for activities such as gesturing, writing and collaborative interaction.

Notwithstanding the potential value of deploying dynamometers to tap into people’s intermediate uncertainty in collaborative situations, we would argue that the most straightforward yet informative way to obtain a valid, nonreactive measure of fluctuating states of uncertainty during collaborative task performance involves analysing the dynamic use of language by team members. For example, tracking and analysing the dialogue arising between team members working on a joint task could provide an exciting window into moments of both individual uncertainty as well as emergent uncertainty at the level of the whole team. We will consider the value of language markers of metacognitive states in the remaining sections of this paper, as we reflect more deeply on the potential to develop a collaborative metareasoning framework that captures the nature of team-based metacognitive monitoring and control.

## 5. Metareasoning in Teams

As discussed above, we contend that one of the most striking and important limitations of the existing metareasoning framework relates to its sole focus on individuals. In this respect, the framework, as currently formulated, gives no consideration to the fact that much real-world reasoning takes place in situations that involve others, such as reasoning that is in the service of cooperative or competitive goals, which themselves are underpinned by many processes, including coordination, conflict resolution, deception, argumentation and persuasion. Indeed, the persuasive function of everyday reasoning is a central tenet of the theoretical approach advanced by Mercier (2016), which is focused on the critical role that is played by argumentation in social communication. Mercier (2016; see also Mercier and Sperber 2011) argues that human reasoning has evolved to enable people to devise arguments and justifications such that they can reap the benefits that come from persuading others. However, the issue of how metareasoning might function in situations that relate to persuasion does not appear to have been considered in any detail to date.

Along with situations that involve persuading others, another context in which reasoning does not operate in isolated individuals is that which is concerned with the attainment of shared or mutual goals, such as the achievement of coordinated joint action or the generation of solutions to complex problems that require collaboration between members of heterogeneous teams. Examples of such team-based reasoning and problem-solving situations are numerous in real-world professional practice. Many of these situations are focused on creative endeavours, such as design, innovation, entrepreneurship, advertising and scientific discovery, yet there are other contexts that are also important, as we have mentioned earlier, such as ones relating to defence, security and emergency response. Overall, there remains a dearth of research that has directly investigated metareasoning in these various kinds of collaborative contexts, such that our current understanding of the nature and operation of metareasoning processes in real-world collaborative activities is highly inadequate.

Notwithstanding the generally limited empirically based understanding that exists of collaborative metareasoning, we note that one of the few team-based domains that *has* been subjected to at least some degree of investigation from a metareasoning perspective is that of real-world design, where several studies have examined the monitoring and control processes that arise when designers are developing creative concepts for new products (for reviews of this literature, see Ball and Christensen 2019, and Richardson et al. 2024). A striking observation from this latter body of research relates to the way in which changes in processing are found to co-occur with the appearance in dialogue of hedge words that reflect uncertainty among team members. For example, when faced with uncertainty, designers often appear to be triggered to engage in “analogical reasoning”, during which they draw upon conceptual ideas from a domain that is different to that of the problem focus and map these ideas across to the current domain (Ball and Christensen 2009; Ball et al. 2010). Likewise, uncertainty that arises in design contexts also seems to evoke “mental simulation”, whereby designers enact or “run” a sequence of interdependent events in a dynamic mental model to determine cause–effect relationships (e.g., between solution components) to predict likely outcomes (Ball and Christensen 2009; Ball et al. 2010; Christensen and Schunn 2009).

Ball and Christensen (2009, 2019) have proposed that cases of analogical reasoning and mental simulation in team design reflect *strategies* that are under metacognitive control, being triggered by emerging uncertainty in the team regarding how to progress toward a design solution. When deployed, these strategies support ongoing design progress while also reducing uncertainty within the team to baseline levels. Outside of the design domain, Chan et al. (2012) have corroborated the existence of a close temporal coupling between uncertainty and the use of analogical reasoning in the context of team-based scientific problem solving. In their research, Chan et al. revealed how expressed uncertainty in team dialogue (similarly indexed through the presence of hedge words) tended to increase prior to episodes of analogical reasoning, subsequently staying at a high level during the analogising process, then returning to a baseline level just after the analogising had terminated. Heightened uncertainty in design teams has also been closely associated with strategic episodes of “problem–solution coevolution” (Wiltschnig et al. 2013). This occurs when designers *simultaneously* refine both their understanding of the design problem and their ideas relating to potential solutions, such that problem understanding informs solution development, as well as vice versa (Dorst and Cross 2001). Wiltschnig et al. (2013) demonstrated that this kind of problem–solution coevolution was more likely to arise during heightened uncertainty in the team-based dialogue and was also linked to increased analogical reasoning.

This research on collaborative metareasoning in team design highlights the critical role that dialogue plays in team interaction to enable teams to make effective control decisions and maintain progress with their design activity. Importantly, such overt dialogue (such as comments that express uncertainty) is not only discernible to interlocutors within collaborating teams but is also visible to researchers interested in investigating the monitoring and control processes that underpin both successful and unsuccessful team activity. In such situations, not only do metacognitive monitoring processes need to be alert to the shifting uncertainty that is salient in the communication of team members, but metacognitive control processes also need to affect dynamic strategy selection and strategy change in a highly coordinated manner if there is to be any hope of problem-solving success. We reiterate, however, that, to date, there has been little direct investigation of such “collaborative metareasoning” in team-based activity. We contend that this lack of research is, in large part, a consequence of the methodological challenges that arise when investigating the dynamic interplay between multiple interlocutors and their associated metareasoning processes.

## 6. Toward a Framework of Collaborative Metareasoning

Understanding the nature of cooperative joint activities has for a long time presented an ongoing challenge for cognitive science. Almost always, the default research approach is to analyse thought and behaviour at the level of the individual, and this is no different in the field of metareasoning. Yet, in team contexts, successful collaboration depends upon joint action and shared understanding between interlocuters. As we noted in our discussion of the metareasoning of individuals, one of the primary cues to judgments of certainty and uncertainty in relation to task-based progress is that of processing fluency; that is, the ease of generating a response. In collaborative contexts, such processing fluency has both an individual dimension, arising as part of each team member’s cognitive processing, and an interpersonal dimension, arising at the level of joint processing. Interpersonal fluency is likely to rely on a variety of cues, some of which will be unique to the interpersonal context. Examples of such cues include background knowledge, familiarity with other team members, perceptions of trust and competence as well as perceived confidence in other team members. Successful team reasoning therefore requires team members to monitor both the individual and joint dimensions of fluency at various points in time, with such dynamic monitoring having the potential to have an impact on procedural decisions to continue with a current processing approach, to switch to a different strategy or potentially to terminate processing if a good solution is not forthcoming.

The importance of team members being able successfully to monitor interpersonal fluency brings to the fore a key issue for research on collaborative metareasoning, which is to investigate and understand the role of alignment and misalignment at this interpersonal level. For example, people tend to find interactions easier and more enjoyable when they like and know other team members, regardless of their individual or joint success with the problem at hand (Richardson et al. 2019). Alignment in interpersonal fluency may play a crucial role in team perceptions of progress on a given task, and hence is likely to be influential in procedural decisions to carry on without making strategy changes. What is generally missing from existing theorising, however, is a model that refers to the interrelationships between individuals in navigating interpersonal fluency from the perspective of the whole team rather than just from the perspective of the individual members who make up the team. As noted, such a model would need to capture the way in which interpersonal fluency is monitored by team members as well as the impact of ongoing fluency dynamics on team members’ control processes in determining strategic decision making.

We suggest that moving from metareasoning considerations at the individual level to considerations at the social and interpersonal level, whether reasoning is serving competitive goals (as in persuasion or deception) or cooperative goals (as in collaborative reasoning and problem solving), will require very careful and highly systematic augmentation of current models of metacognition, such as Ackerman and Thompson’s (2017, 2018) metareasoning framework. Even a cursory assessment of how metareasoning might take place in team-based reasoning indicates the complexity of the theoretical issues that immediately become foregrounded. As we have noted, what is particularly fascinating, yet poorly understood, is the way in which individual members of teams invoke and coordinate both individual-level and team-level metacognitive monitoring and control processes to drive forward their joint action and strategic decision making. The metacognitive monitoring and control processes that team members need to engage will presumably have to be attuned to the subtleties and complexities of both personal and interpersonal uncertainty, with the latter most likely being made manifest in the verbal and nonverbal communication arising during team interaction.

As mentioned in our introduction, an important tripartite distinction can be drawn in relation to collaborative metareasoning between “self-monitoring” (i.e., an individual’s perception of their own performance), “other monitoring” (i.e., an individual’s perception of the performance of others) and “joint monitoring” (i.e., the unified perception of collective performance), as articulated by Richardson et al. (2024; see also Pickering and Garrod 2021). When considering this tripartite distinction specifically in relation to fluctuating states of certainty in reasoning contexts, it can be seen how the distinction gives rise to concerns not only with an individual’s own feeling of confidence in a solution to a reasoning task but also that individual’s awareness of a collaborator’s feeling of confidence in this solution, as well as the collective or agreed-upon confidence in the solution. All these elements of ongoing monitoring have the potential to impact decisions about task progress in important ways and are critical in informing a shared understanding of the reasoning task at hand. As we also noted earlier, a parallel tripartite distinction can be drawn that relates to control processes, which can be captured by the notions of “self-focused control” (i.e., an individual’s decisions about how to progress or terminate their own reasoning), “other-focused control” (i.e., an individual’s decisions about how to control the performance of others) and “joint control” (i.e., the unified control of decisions regarding how to advance or terminate collective performance).

Table 1 presents a more detailed characterisation of key monitoring and control processes that arise across the three proposed levels of metacognition, as already discussed above and further elaborated upon in the next section. The table also summarises examples of the kinds of cues that are very likely to be detected by monitoring processes, as well as examples of the kinds of outcomes that will result from the deployment of control processes. We contend that it is possible to track many of the key monitoring and control processes associated with our tripartite distinction through an analysis of ongoing dialogue (Gonzales et al. 2010; Richardson and Nash 2022). Through such analyses, it is possible, for example, to identify periods of conflict (Tausczik and Pennebaker 2013) or uncertainty within team reasoning, problem solving and decision making (e.g., as reflected in the use of tentative language or hedge words), as well as strategic changes that arise as a result of the detection of conflict or uncertainty. We therefore propose that research on collaborative metareasoning would benefit from the development of theoretical models that capture how uncertainty, as well as both alignment and misalignment, can trigger self- and group reflection and downstream strategic decision making. Importantly, we suggest that these periods of uncertainty can be tracked dynamically by measuring social signals, specifically the linguistic, paralinguistic and proxemic features that occur during dialogue between interlocuters.

## 7. The Role of Language in Collaborative Metareasoning

In dialogue, speakers process a great deal of information, they take and give the floor to each other as well as plan and adjust their contributions on the fly. Despite the level of cognitive effort and control that it requires, dialogue is the easiest way speakers possess to come to similar conceptualisations of the world. For example, Pickering and Garrod (2021) suggest that it is the process of alignment, a largely automatic and unconscious process whereby speakers use language in the same way to reach mutual understanding, that underpins many successful joint activities.

Research on alignment indicates that, over time, in successful communication, people tend to think in the same way (representing the world in the same way) and use similar terms of expression. For example, in problem solving, speakers align with each other by mutually controlling the flow of dialogue and by constantly monitoring their own and others’ ways of representing information. Furthermore, alignment increases over time as the dialogue progresses. In a verbal description task, reported by Clark and Wilkes-Gibbs (1986), participants in the role of “director” started describing a figure to a “matcher” using long and detailed sentences (e.g., “the next one looks like a person who’s ice skating, except they’re sticking two arms out in front”). In subsequent turns, descriptions became simpler (e.g., “the fourth one is the person ice skating, with two arms”), until the interlocutors converged on a common description (“the ice skater”) and used it effectively until the end of the task. What this means is that speakers gradually converge on a similar conceptualisation of the task (termed “situation model alignment”; Garrod and Anderson 1987) by aligning at a linguistic level (termed “linguistic alignment”).

Alignment at the level of joint monitoring and control can be understood in terms of the important theoretical concept of “intersubjectivity”, which refers to having a shared understanding of an object (e.g., Mori and Hayashi 2006) or an interlocuter (Gillespie and Richardson 2011). Like alignment, intersubjectivity is frequently thought of as being an implicit and automatic behavioural orientation toward others (Coelho and Figueiredo 2003; Merleau-Ponty [1945] 1962). For example, frequent, brief, paralinguistic communications such as “uh-huh” or head-nodding that punctuate a conversation serve a purely social function. They are not designed to contribute additional meaning but are instead used to provide ongoing feedback about comprehension (Schegloff 1982), thereby signalling implicit understanding to other team members. A similar role is played by the “third position repair” described by Schegloff (1992), whereby a listener will often respond to a speaker with an utterance which, instead of contributing anything new, simply displays understanding of what has been said.

Although research typically suggests that intersubjectivity and alignment are key to successful collaboration, Richardson et al. (2007) additionally propose that misalignment (or what they refer to as “disalignment”) is also likely to trigger a solution to a team-based problem. Such misalignment is similar to situations in which recognition of uncertainty can trigger a strategy change that moves the task towards successful resolution (Ball and Christensen 2009, 2019). For example, misalignment can lead to teams engaging in conflict-resolution attempts that can promote team agreement on strategy change. The benefits for team-based reasoning that can derive from misalignment have been demonstrated in a study reported by Paletz et al. (2017), in which they analysed the temporal relationships between brief interpersonal disagreements (or “microconflicts”) and the subsequent expression of uncertainty in conversations arising in successful and unsuccessful engineering product design teams. Paletz et al. discovered that microconflicts were followed by a relative decrease in uncertainty in successful design teams, whereas uncertainty increased after microconflicts in unsuccessful design teams. Paletz et al. (2017) interpret these findings as suggesting that the interaction between conflict and uncertainty may be critical in determining the success of design teams.

Building on the idea of the importance of misalignment and uncertainty for task progress, we further note here that Bjørndahl et al. (2015) suggest that agreement between interlocuters is not enough when working toward a solution to a problem. They identify three key interactional styles, one of which they refer to as an “integrative style”, which is characterised by self–other repair via, for example, clarification requests, disagreements, questions and explicit negotiation of ideas and proposals. During a collaborative LEGO modelling task, Bjørndahl et al. (2015) found that this integrative style, which promotes explicit miscommunication, generated more innovative models as compared to an inclusive praise-based style or an instructional self-repair style. Crucially, speakers do not reach alignment in isolation, but through interaction, by manipulating each other’s contributions. Furthermore, speakers are able to track this alignment by metarepresenting it (Gandolfi et al. 2023). Speakers mutually control the flow of dialogue and constantly monitor their own and their interlocutors’ way of representing information.

What is missing from the existing literature on metareasoning is a framework that captures metareasoning in teams, and that offers a means of dynamically tracking key processes, such as fluency and uncertainty, between team members. Our framework (see Table 1) proposes tripartite distinctions relating to both metacognitive monitoring and control that can be meaningfully applied to understand collaborative metareasoning at the level of self-, other and joint processing, while also focusing on how collaborative metareasoning unfolds via dialogue between team members. We argue that the perception of misalignment triggers monitoring, which leads to negotiation between perspectives. During collaborative monitoring, for example, alignment can be seen as a process that requires the combination of metacognition (i.e., with respect to oneself) and social cognition (i.e., with respect to interlocutors, Gandolfi et al. 2023).

Team members not only predict and monitor the utterances of interlocutors, but they also predict and monitor their own utterances, as well as acting on joint representations by comparing their expectation of what their interlocutors might say, with what they actually say. When discrepancies arise between what is predicted and what arises, this typically leads to subsequent reformulations, expansions or clarifications that help drive the interaction forward. Specifically, when speakers metarepresent the failure of alignment they will reformulate their plans and correct their contributions to keep the dialogue on track.

Control is especially necessary for the attainment of joint reasoning activities and team decision making. We also argue that control plays out via the interaction between interlocuters, who do not reach alignment in isolation, but do so by manipulating each other’s contributions in a collaborative fashion. By continuously monitoring and comparing self and others’ contributions, and specifically by metarepresenting whether they believe they and their interlocutor are aligned or not, team members make decisions about whether to continue a particular course of action, terminate the task or switch strategies to find an alternative way forward.

## 8. Conclusions and Future Directions

A key focus in this paper has been on the importance of analysing the use of language when examining collaborative metareasoning. For example, diminishing confidence, increasing interpersonal misalignment and emerging conflict—as revealed through language change during team reasoning—appear to be linked to metacognitive control decisions to adopt new approaches or to terminate processing (cf. Paletz et al. 2017). These dynamic and objective language-based measures of unfolding metareasoning processes in teams can also be compared with more traditional self-report measures, discussed above, that relate to task performance, solution confidence and interpersonal dynamics. In addition, sophisticated approaches to language analysis in teams (Bjørndahl et al. 2015) can be invaluable for identifying important aspects of behavioural sequencing, as well as for uncovering clusters of behaviours that capture the team-based monitoring of ongoing processing fluency and disfluency.

We suggest that future research examining language use in teams as an index of metacognitive monitoring and control could explore the way in which the different interactional styles of team members modulate metareasoning processes. In this respect, we note that Gonzales et al. (2010) have shown that, in groups with a strong hierarchy, an authoritarian leadership style is often characterised by the frequent use of self over collective pronouns, and that this display of pronouns can be detrimental for team cohesion. We contend that the impact of different leadership styles is also likely to manifest in unique ways in the dialogue that relates to joint metacognitive monitoring and control during team reasoning. These modulating effects of leadership styles remain to be examined empirically, yet are clearly important to explore in high-stakes, real-world, decision-making contexts where effective team reasoning is critical.

We also propose that our collaborative metareasoning framework could be extended to take into account multimodal measures of team interaction. For example, the fine-grained analysis of real-time social signals could provide further valuable insights into fluctuating levels of confidence and cohesion in teams in relation to ongoing monitoring and control processes (cf. Casakin et al. 2015). Indeed, although we have emphasised the way in which an analysis of language can provide valuable information about ongoing monitoring and control processes in team-based reasoning, this is not to dismiss the insights into collaborative metareasoning that might also be gained from other forms of behavioural analysis. In this respect, we acknowledge the existence of substantial bodies of behavioural research relating to the complex interplay that arises between individuals engaged in the achievement of common goals, including studies concerning the nature of joint action (e.g., Sebanz and Knoblich 2009), collective intelligence (e.g., Krause et al. 2010), synchrony (e.g., Lakens and Stel 2011; Miles et al. 2011), perspective taking (Gillespie and Richardson 2011) and distributed cognition (e.g., Hutchins 1995). A detailed review of this literature might well pinpoint important findings that could be informative about collaborative metareasoning. What seems more likely, however, is that research in these areas—apart from studies of synchrony and perspective taking—has typically been more concerned with the nature of people’s task-oriented, object-level processing (e.g., coordinated action or team decision making) than with people’s metareasoning processes. Nevertheless, until these other research areas are carefully examined through a metareasoning lens, it remains possible that important knowledge is being missed.

What also seems clear is the need for our proposed collaborative metareasoning framework to be evaluated and extended through extensive empirical research. Indeed, the tripartite distinction that we articulate relating to self-, other and joint metareasoning only presents a starting point for further conceptual development regarding the nature of these metacognitive processes and their interconnections. In this respect, we concede that the literature on collaborative metareasoning is simply not sufficiently well advanced to enable much in the way of detail and precision concerning the highly complex interrelationships that are certain to exist between individual metareasoning and collaborative metareasoning processes. More positively, it does seem that major conceptual advancements are increasingly likely as interest in metareasoning continues to grow (e.g., see De Neys 2023).

What is also encouraging for the ongoing development of the field of collaborative metareasoning is the potential for a multimethod investigative approach to be able to elicit highly rich and informative data regarding the nature and interplay between individual and collaborative metareasoning processes. Such a multimethod approach can, for example, lend itself to the identification of key subjective metrics (e.g., self-report measures that index trust and confidence in oneself and in others) as well as objective metrics (e.g., language-based markers of uncertainty and eye-gaze measures to pinpoint challenges arising in joint cognition). Empirical research additionally needs to track processes of alignment and misalignment via dialogue (Richardson and Nash 2022) to identify periods of conflict or uncertainty within reasoning (e.g., as reflected in the use of tentative language, hedge words or pronouns), and compare these indices with measurements of traditional metareasoning concepts (e.g., fluency, intermediate confidence and strategy change).

We contend that the conceptual progress that can derive from our proposals for a collaborative metareasoning framework can additionally provide valuable opportunities to develop interventions that might support enhanced reasoning, problem solving and decision making in real-world contexts, including those that we identified throughout our paper (e.g., ones relating to design, innovation, defence, security, surveillance and emergency response). Interventions targeted at supporting metareasoning might assist, for example, with the identification of periods of uncertainty, points of impasse or instances of misunderstanding, which can then prompt teams to reflect on more effective strategic choices. Such metareasoning support tools would represent an original and radical approach to facilitating successful collaboration in applied contexts, augmenting natural reasoning and metareasoning processes in useful ways that teams could capitalise upon.

We have attempted here to advance the metareasoning literature by commencing the development of an understanding of the metareasoning processes that arise in team-based contexts. We propose a collaborative metareasoning framework that emphasises the importance of a tripartite distinction between self-, other and joint metareasoning for capturing the different levels of monitoring and control that arise during task-based processing. We also suggest that the interplay that occurs between different levels of metacognitive monitoring and control is critical for a team’s procedural decision making, such as collective decisions to accept an initial solution, to switch strategy or to give up as well as to a team’s potential achievement of a successful task outcome. We additionally argue for the utility of analysing language as a means of tracking the fluctuating states of uncertainty and misalignment that arise, with such states seemingly having the capacity to act as metacognitive triggers for strategy change. In conclusion, our framework begins to address a significant gap in the literature surrounding the monitoring and control processes that play out during collaborative reasoning, whereby complex, interpersonal interactions occur as people work together to achieve shared goals.

## Figures and Tables

**Table 1:** A detailed characterisation of indicative monitoring and control processes that arise across three proposed levels of metacognition. The table also summarises examples of the cues that are likely to be detected by monitoring processes, as well as examples of the outcomes of control processes.

Levels of Monitoring	Indicative Monitoring Processes	Example Cues Detected by Monitoring Processes
**Self-Monitoring** An individual’s perception of their own performance.	**An individual’s generation of an:** initial judgment of solvability; feeling of rightness; feeling of error; feeling of warmth; intermediate confidence or uncertainty; final confidence; final judgment of solvability.	**An individual’s sensitivity to:** processing fluency (ease of processing); perceived features of the presented task; perceived task complexity; study time; response time.
**Other Monitoring** An individual’s perception of the performance of others.	**An individual’s perception of someone else’s:** initial judgment of solvability; feeling of rightness; feeling of error; feeling of warmth; intermediate confidence or uncertainty; final confidence; final judgment of solvability. **An individual’s perception of:** alignment/misalignment.	**An individual’s sensitivity to someone else’s:** processing fluency (ease of processing); perceived features of the presented task; perceived task complexity; study time; response time; degree of agreement; level of understanding (potentially made manifest by language markers such as hedge words and pronoun use).
**Joint Monitoring** The unified perception of collective performance.	**A group’s unified perception of:** initial judgment of solvability; feeling of rightness; feeling of error; feeling of warmth; intermediate confidence or uncertainty; final confidence; final judgment of solvability. **A group’s perception of:** alignment/misalignment.	**A group’s unified perception of:** processing fluency (ease of processing); perceived features of the presented task; perceived task complexity; study time; response time; degree of agreement; level of understanding (potentially made manifest by language markers such as hedge words and pronoun use).
**Levels of Control**	**Indicative Control Processes**	**Example Outcomes of Control Processes**
**Self-Focused Control** An individual’s decisions about how to progress or terminate their own reasoning.	**An individual’s procedural decision to engage in:** memory search; reasoning, problem solving or decision making; response generation; response evaluation; strategy change; giving up; help-seeking.	**An individual’s generation of:** recalled information; an intermediate or final response (e.g., a solution, option or decision, including the decision to give up); an evaluation of an intermediate or final response; a new process or strategy (e.g., analogising, mental simulation); a request for help.
**Other-Focused Control** An individual’s decisions about how to control the performance of others.	**An individual’s procedural decision to engage in:** affirmation; encouragement; persuasion; argumentation; negotiation; manipulation; deception.	**An individual’s generation of:** alignment (e.g., situation model alignment and linguistic alignment); intersubjectvity; shared understanding; common ground; conflict resolution; misalignment; disalignment.
**Joint Control** The unified control of decisions regarding how to advance or terminate collective performance.	**A group’s unified procedural decision to engage in:** memory search; reasoning, problem solving or decision making; response generation; response evaluation; strategy change; giving up; help-seeking.	**A group’s unified generation of:** recalled information; an intermediate or final response (e.g., a solution, option or decision, including the decision to give up); an evaluation of an intermediate or final response; a new process or strategy (e.g., analogising, mental simulation); a request for help.

## Data Availability

No new data were created or analyzed in this study. Data sharing is not applicable to this article.
